# Gut Microbiome Metagenomics Analysis Suggests a Functional Model for the Development of Autoimmunity for Type 1 Diabetes

**DOI:** 10.1371/journal.pone.0025792

**Published:** 2011-10-17

**Authors:** Christopher T. Brown, Austin G. Davis-Richardson, Adriana Giongo, Kelsey A. Gano, David B. Crabb, Nabanita Mukherjee, George Casella, Jennifer C. Drew, Jorma Ilonen, Mikael Knip, Heikki Hyöty, Riitta Veijola, Tuula Simell, Olli Simell, Josef Neu, Clive H. Wasserfall, Desmond Schatz, Mark A. Atkinson, Eric W. Triplett

**Affiliations:** 1 Department of Microbiology and Cell Science, University of Florida, Gainesville, Florida, United States of America; 2 Department of Statistics, University of Florida, Gainesville, Florida, United States of America; 3 Department of Clinical Microbiology, University of Kuopio, Kuopio, Finland; 4 Hospital for Children and Adolescents, University of Helsinki, Helsinki, Finland; 5 Department of Virology, Tampere University Hospital, Tampere, Finland; 6 Department of Pediatrics, University of Oulu, Oulu, Finland; 7 Department of Pediatrics, Turku University Hospital, Turku, Finland; 8 Department of Pediatrics, University of Florida, Gainesville, Florida, United States of America; 9 Department of Pathology, Immunology and Laboratory Medicine, University of Florida, Gainesville, Florida, United States of America; East Carolina University School of Medicine, United States of America

## Abstract

Recent studies have suggested a bacterial role in the development of autoimmune disorders including type 1 diabetes (T1D). Over 30 billion nucleotide bases of Illumina shotgun metagenomic data were analyzed from stool samples collected from four pairs of matched T1D case-control subjects collected at the time of the development of T1D associated autoimmunity (i.e., autoantibodies). From these, approximately one million open reading frames were predicted and compared to the SEED protein database. Of the 3,849 functions identified in these samples, 144 and 797 were statistically more prevalent in cases and controls, respectively. Genes involved in carbohydrate metabolism, adhesions, motility, phages, prophages, sulfur metabolism, and stress responses were more abundant in cases while genes with roles in DNA and protein metabolism, aerobic respiration, and amino acid synthesis were more common in controls. These data suggest that increased adhesion and flagella synthesis in autoimmune subjects may be involved in triggering a T1D associated autoimmune response. Extensive differences in metabolic potential indicate that autoimmune subjects have a functionally aberrant microbiome. Mining 16S rRNA data from these datasets showed a higher proportion of butyrate-producing and mucin-degrading bacteria in controls compared to cases, while those bacteria that produce short chain fatty acids other than butyrate were higher in cases. Thus, a key rate-limiting step in butyrate synthesis is more abundant in controls. These data suggest that a consortium of lactate- and butyrate-producing bacteria in a healthy gut induce a sufficient amount of mucin synthesis to maintain gut integrity. In contrast, non-butyrate-producing lactate-utilizing bacteria prevent optimal mucin synthesis, as identified in autoimmune subjects.

## Introduction

As the incidence of type 1 diabetes (T1D) in developed countries has been increasing at a rate far beyond the rate of population growth, environmental factors have been considered as likely candidates responsible for this change in disease incidence in recent decades [1]–[3]. Of those factors, the gut microbiota have come under recent interest; supported in part by observations in both non-obese diabetic (NOD) mice and BioBreeding Diabetes Prone (BB-DP) rats where antibiotic use prevents the onset of diabetes [4], [5]. Initial studies found that NOD mice raised in germ-free environments would spontaneously develop diabetes [6], while a recent study suggested that rather than being diminished under germ-free conditions, the development of T1D can be prevented through modulation of the intestinal microbiota [7]. Likewise, both the NOD mouse and BB-DP rats treated with Freund's adjuvants or *Lactobacillus* strains delayed or decreased incidence of diabetes [8]–[16]. With respect to mechanisms of action, the gut microbiome of NOD mice lacking an adaptor for multiple innate immune receptors responsible for recognizing microbial stimuli correlates with disease onset, revealing a relationship between gut microbiota and the immune system [15].

To explore specific differences in the microbial communities responsible for T1D modulation, 16S rRNA amplicons were sequenced from BB-DP and BioBreeding Diabetes Resistant (BB-DR) rat stool samples collected around the time of diabetes onset [17]. This analysis revealed bacteria genera whose members were either positively or negatively correlated with diabetes. *Lactobacillus* and *Bifidobacterium* were more abundant in BB-DR rats while *Bacteroides* and *Clostridium* were more abundant in BB-DP rats. Both *Lactobacillus* and *Bifidobacterium* are well known to have members with probiotic characteristics.

In humans, inflammatory bowel diseases (IBD) such as Crohn's and ulcerative colitis, are thought to be autoimmune and have been correlated with a depletion of commensal bacteria from the phyla Firmicutes and Bacteroidetes [18]. Fecal microbial communities in identical twins with Crohn's disease show a decrease in diversity when compared to healthy twins; likewise, the microbial communities of the twins with Crohn's disease were less similar to one another than the communities from the healthy individuals [19]. Evidence from IBD studies indicates that gut microbiota are responsible for driving inflammatory responses regulated by regulatory T cells. Without regulatory T cells, commensal gut microbiota can erroneously stimulate an inflammatory response and cause IDB [20]. Autoimmune diseases such as multiple sclerosis, rheumatoid arthritis, psoriasis, and T1D have all been related to an increase in Th1/Th17 as the result of an elevated or uncontrolled immune response [21].

With respect to human T1D, the proportion of bacteria within the phylum Bacteroidetes increases over time in unhealthy (i.e., autoantibody positive, “autoimmune”) subjects while in healthy non-T1D prone subjects, the proportion of the Firmicutes within the population increases [22]. In addition, the case samples within that study were less diverse than the control samples, and case microbiomes had lower Shannon diversity compared to those in individuals who progressed towards autoimmune T1D [22]. Over time, case microbial communities became increasing less similar to each other compared to the control communities who were more similar to each other. Taxonomic identity provides information regarding the microbial communities, but falls short of expressing the functions present in the environment. To determine the functions that define the autoimmune microbiome, metagenomics analysis was needed and forms the subject of this report.

## Methods

### Ethics statement

This research has been approved in writing by the Institutional Review Board of the University of Florida. The analysis of the stool samples was done without knowledge of the identity of the human subjects. The Diabetes Prevention and Prediction study group in Finland obtained informed consent from the parents of the subjects involved for the collection of the stool samples. Written consent was obtained from the parents to collect these samples.

The samples used in this study came from eight Finnish children described previously [22]. Fecal samples were taken after two autoimmune antibodies were identified in the four case samples, and at corresponding time points in controls. The children were age matched and were all HLA-DQ genotype positive. DNA extraction was performed as described previously [23] and sequencing was conducted by Argonne National Laboratory. The original sequences are submitted to GenBank as study accession number SRA036573.1.

The metagenome library produced paired reads with an overlap region of approximately 30 bases. Paired sequences were assembled using a custom script, stitch.py. Sequences that were not paired were quality trimmed using default CLC Genomics Workbench version 4.0 (CLC) parameters. Reads less than 50 bases in length were discarded.

The average genome size of each sample was calculated using a recently described normalization method [24] whereby the metagenome data is mined for the highly conserved, single copy genes *rpl*A, *rpl*D, *rpo*B, *rps*J, *rpl*C, *rpo*A, *rps*G, and *rps*Q. Genome size calculations are made on the basis of the abundance of these genes found within each metagenomic sample.

Comparisons of functions determined directly from contigs using BLASTX revealed highly similar microbiomes at the function level. However, because this analysis was only conducted at the contig level, information was only provided about the presence or absence of a particular function. Because of this restriction, little could be done to compare between samples. It is speculated that this is primarily as a result of the depth of sequencing in which even low abundant genomes are sequenced and their DNA fragments are assembled into contigs. Since the gut microbiomes in the study are comprised of highly similar functions, it was determined that a qualitative approach was needed in order to resolve differences between the metagenomes.

Rather than making direct comparisons of contigs to sequenced genomes, in which the prevalence of particular functions cannot be determined, a quantitative approach was taken in which each contig was used as the basis for ORF prediction using the Prodigal algorithm, followed by an assessment of the coverage of each ORF.

Prodigal was run using a metagenome setting in which pre-created training files are used to make the predictions. The output of the algorithm includes both the nucleotide sequences and their amino acid translations. The nucleotide sequences were used to determine the coverage of each ORF by mapping reads back to them using the same parameters that were used during de novo assembly. The number of reads mapped to an ORF correlates to the prevalence of the function within the genomes of the microbiome. The amino acid sequences were used for functional annotation using phmmer.

Phmmer conducts alignments of amino acid sequences to amino acid databases much like BLASTP does. However, unlike BLASTP, phmmer uses a hidden Markov model to predict protein domains, thus affording the algorithm greater specificity and a lower rate of false positives. The SEED database was chosen for the ORF alignments because of its subsystem structure. With the goal being to group functions found within the microbiomes into hierarchical structures, in order to determine if a particular subsystem or subsystem hierarchy (in addition to function) is more prevalent in cases or controls, the SEED database was filtered for references with subsystem information.

Having established the abundance of each function and functional hierarchy based on the number of reads, it is possible to make a quantitative comparison between the abundance of functions within the microbiomes. However, due to the large number of sequences representing each microbiome, and the relatively small counts within each function, statistical methods such as chi-square are unable to determine significance. To overcome this, a Poisson model was used.

De novo assemblies were conducted using a global CLC alignment with parameters dependent on read length. The most stringent cost values for mismatches, insertions, and deletions were used. A minimum contig length of 400 bases was required. Open reading frames were predicted from contigs produced from the de novo assemblies using the metagenome implementation of Prodigal v.2.0 (citation). To determine the coverage of each ORF, CLC reference assemblies were conducted to the nucleotide sequences of each ORF using the parameters used during de novo assembly.

Functions were assigned to predicted ORFs by protein alignments with phmmer v.3.0 (http://hmmer.org/) and a version of the SEED database downloaded in August 2010 and modified to included only sequences with subsystem annotations. Only alignments with E-values less than 10^−6^ were considered for functional assignment. The best phmmer hit for each ORF was selected by choosing the match with the highest full-sequence bit-score. The full-sequence bit-score is calculated by summing the bit-scores for each protein domain, and the individual domain bit-scores are calculated based on a hypothetical envelope of where the domain is located on the protein, based on the hidden Markov model of the phmmer algorithm.

To compare the metabolic potential of case and control metagenomes, each functional hierarchy and function defined by the SEED subsystems database was quantified by the number of reads in each sample for that function.

The ORFs that did not have significant matches to the SEED subsystem database were combined and their amino acid sequences were clustered using CD-HIT v.4.3 and a 40% sequence identity threshold. The corresponding nucleotide translations for the sequences from each cluster were used to determine the coverage of each cluster, in each sample, using CLC and the reference assembly parameters that were used for functional ORF coverage analysis.

**Figure 1:**
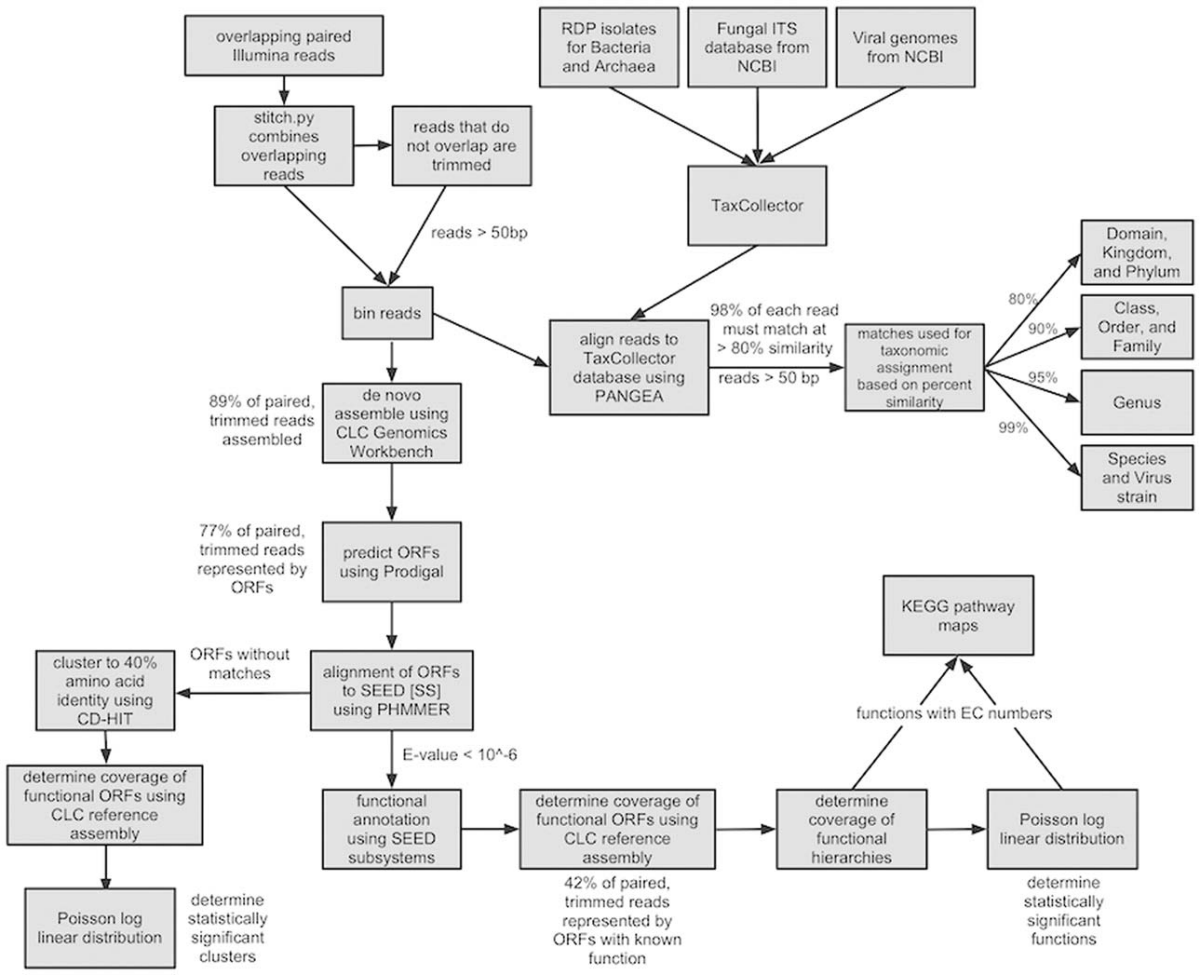
The pipeline of metagenome sequence analysis used in this work.

Taxonomic assignment was conducted on all reads over 50 bases long. Classification was determined through reference assemblies to an RDP database containing sequences for Archaea, Bacteria, and Eukaryota, modified by TaxCollector [25]. RDP and viral genome databases were downloaded in February 2011, and a fungal ITS database in September 2010. The assemblies were conducted using CLC at 98% length fraction with similarity cutoffs that varied based on the taxonomic level being analyzed (Figure 1).

The number of reads in each functional hierarchy, function, cluster, and OTU were used in a Poisson log-linear regression model to identify categories with significantly different read counts between cases and controls. A p-value of 0.01 or less was chosen as the criterion for statistical significance.

KEGG maps were drawn based on statistically significant functions annotated by SEED subsystem database entries with enzyme commission (EC) numbers. Principal component analysis (PCA) was conducted on abundance measures for SEED functions and OTUs. For both SEED functions and OTUs, the raw counts were standardized by dividing by the total number of reads for each sample, and the subsequent fractions were log transformed. After log transforming the SEED function abundances, the data were centered using the formula found in the supplemental materials. OTUs were first filtered by significance according to the Poisson model (OTUs were required to have a p-value of 0.01) before PCA was performed. The calculations for PCA were done using the R “prcomp” function and plotted using the “plot” function.

The statistical model was fit as a generalized linear mixed model with log link. A subject specific random effect was used to control for the correlation, this is typically called a Poisson regression model with a subject specific random effect. To fit the data, the glmer package in R, under the library lme4, with family = Poisson, was used [26]–[28].

## Results

### Average genome size

To determine whether our analyses might be skewed by a difference in average genome size between cases and controls, the average genome size within the DNA of each sample was determined. No difference in average genome size between the case and control samples was observed using a paired, two-tailed t-test (Fig. S1). The average genome size of the control metagenomes was 2.87±0.21 Mbp while that of the case metagenomes was 2.48±0.18 Mbp. Thus, any quantitative functional differences observed between cases and controls cannot be attributed to any bias related to differences in average genome size.

### Steps in metagenome analysis

The analysis of the metagenomes in this work had four objectives: 1) the calculation of the diversity of predicted proteins in each sample; 2) the quantitative differences in the abundance of functions between cases and controls; 3) the depiction of these differences on KEGG maps, and 4) the mining of highly conserved rRNA genes to determine the taxonomic differences between cases and controls (Fig. 1). A guiding principle of this work was to determine the quantitative differences in the functions of cases and controls by mapping the raw reads back to the predicted ORFs by reference assembly. Those ORFs were identified through the assembly of the data.

The *de novo* assemblies made from these data resulted in contigs representing 89% of total reads after pairing and quality trimming. A minimum contig length of 400 bases was required for the entry of any contig into our database. The largest contig was 444,885 bp. The average contig length was 1,487 bases, and the median length was 634 bases. Prodigal was used to predict an average of 121,523 ORFs per sample, which represented 77% of all paired and unpaired reads. Phmmer alignments to the SEED subsystem database established functional assignment for an average of 118,928 ORFs per sample representing 42% of all reads. All ORFs were classified to 2,887 functions and 959 functional hierarchies.

### Analysis of the metagenomics data

**Figure 2:**
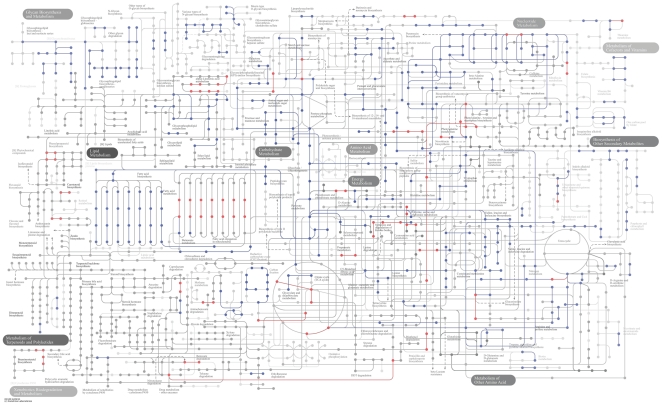
Statistically significant metabolic steps in controls (blue) and cases (red).

According to the Poisson model, 911 functions exhibit a statistically significant difference in prevalence between cases and controls, where 114 were more prevalent in cases and 797 in controls (supporting dataset S1). At the most general functional hierarchy, carbohydrates and stress responses were more prevalent in cases, while 17 hierarchies, mostly in secondary metabolism, were more prevalent in controls (Fig. 2, supporting dataset S1). The Poisson model identifies nearly eight times as many functions with a greater abundance in controls verses cases. These functions are also of much greater abundance than those identified in cases (supporting dataset S1).

Abundances for all functions were used for principle components analysis (PCA) in order to determine the clustering of samples (Fig. S2). PCA reveals that the controls all exhibit similar functionality while the cases all express different functions between each other and the controls. The majority of SEED functions are annotated with EC numbers. These EC numbers can be used to map functions identified with SEED onto KEGG maps (Fig. S3). Of the 1,061 pathways identified in KEGG, 24 and 166 were statistically more prevalent in cases and controls, respectively (Fig. 2). The complete list of functions and subsystems, their abundances, the results of the Poisson model, and their prevalence in either cases or controls, can be found within the supplemental data (supporting dataset S2). Among all of the metagenome samples, CD-HIT clustered 517,636 predicted ORFs with unknown function into 186,005 clusters. Of these, 38,645 have more than 100 reads, and according to the Poisson model, 3,791 exhibit a statistically significant difference in the number of reads between cases and controls.

The metagenomic data was also mined for 16S rRNA. Using reference assembly, all reads were mapped against the TaxCollector-modified RDP database for 16S rRNA [25]. Assuming that there is one 16S rRNA gene (1,400 bases long) for every megabase of DNA sequenced in a metagenome, 19.49 gigabases in the eight metagenomes would contain 19,490 16S rRNA genes and 219,670 16S rRNA reads. Taxonomic assignment based on the 16S rRNA gene assigned 251,058 reads to the domain bacteria and 126,817 reads to known bacterial species. The complete list of operational taxonomic units at six taxonomic levels, their relative abundances, the statistical results, and their prevalence in either cases or controls is available in the supplementary material (supporting dataset S3).

At the phylum level and at p-values less than 0.001, Actinobacteria, Bacteroidetes, and Proteobacteria were higher in cases while Firmicutes, Fusobacteria, Tenericutes, and Verrucomicrobia were higher in controls. With a p-value of 0.01 or less, 161 genera of bacteria were found that differed in abundance between cases and controls (supporting dataset S4). Of these, 79 were higher in cases, with the remaining 82 higher in controls.

**Figure 3:**
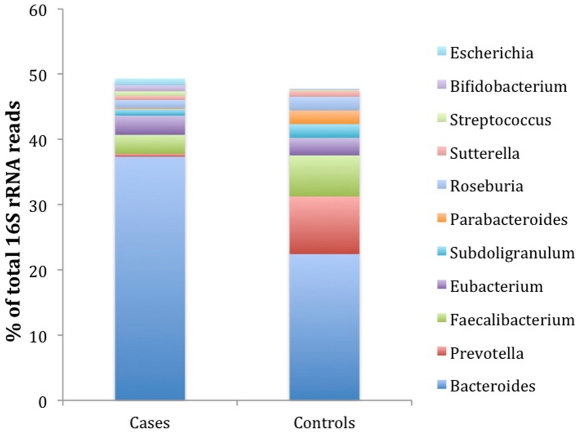
Mean proportion of the 11 most abundant genera that differ significantly between cases and controls (p value≤0.01).

The differences between cases and controls are particularly striking at the genus level. The greatest % differences between cases and controls were in the genera *Prevotella* and *Bacteroides* where *Prevotella* was much more abundant in controls and *Bacteroides* was much higher in cases (Fig. 3, supporting dataset S4). The large decline in *Bacteroides* in controls compared to cases is compensated entirely by Prevotella and the butyrate producers. The butyrate producers include members of the genera *Eubacterium*, *Fusobacterium*, *Anaerostipes*, *Roseburia*, *Subdoligranulum*, *Faecalibacterium*, and other cultured genera, which have not yet been given genus names (supporting dataset S4).

**Figure 4:**
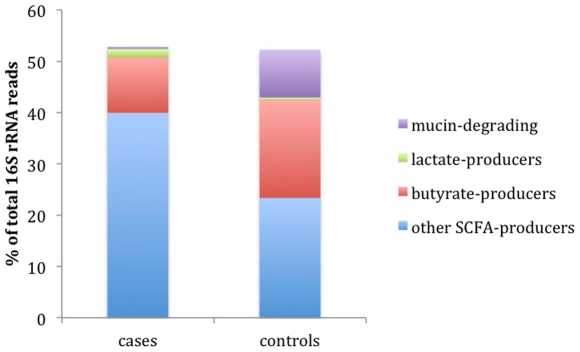
Mean proportion of four functional groups that differ significantly between cases and controls (p value≤0.01). Depicted is the abundance of 16S rRNA reads that are assigned to genera known to produce butyrate, lactate, or other short chain fatty acids (SCFA) such as propionate, acetate, or succinate. Also shown is the proportion of bacteria that degrade mucin.

In contrast to the butyrate producers, the lactate producers, *Lactoabcillus*, *Lactococcus*, *Bifidobacterium*, and *Streptococcus* were more abundant in cases; with the difference between cases and controls being 1.19% of all 16S rRNA reads. Another group of interest is the mucin-degrading bacteria in the genera *Prevotella* and *Akkermansia*. There is a 20- and 140-fold higher proportion of *Prevotella* and *Akkermansia*, respectively, in controls compared to cases. *Prevotella* is the second most abundant genus in controls comprising over 8.8% of all 16S rRNA reads. All of these trends observed at the genus level are also seen at the species level. Other bacteria that compete with the butyrate producers for lactate as carbon source, such as *Veillonella*, produce propionate from lactate fermentation rather than butyrate. *Veillonella* can compete for lactate substrate with the butyrate producers and are in statistically higher abundance in cases than controls. Cases possessed 6.7 times more *Veillonella* than did controls. Other genera known to produce short chain fatty acids (SCFA) other than butyrate, such as *Bacteoides*, and *Alistipes* were proportionately higher in cases than controls. In summary, the 16S rRNA data shows that the increased proportion of bacteria in cases that produce SCFA other than butyrate producers are substituted by a corresponding increase in the proportion of butyrate producers and mucin degraders, in control subjects (Fig. 4).

The functional data support the 16S rRNA results which suggest a higher level of butyrate production in control microbiomes. The proportion of reads that assemble to butyryl-CoA dehydrogenase (EC 1.3.99.2) is significantly higher in controls (p value = 0.0005) than in cases. Butyryl-CoA dehydrogenase catalyzes an important rate-limiting step in butyrate synthesis in *E. coli* [29]. Unfortunately, the known mucin degradation genes [30] are not present in the SEED database used for the discovery of gene functions in these datasets. In addition, the mucin degrading genes have only been described in one organism, making it difficult to mine these genes accurately from metagenomic data.

## Discussion

Our previous work showed taxonomic differences between the gut microbiomes of healthy children compared to autoimmune children in a cohort of samples from Finland [22]. In this work, the objective was to learn the metabolic potential of these bacterial communities by shotgun sequencing DNA extracted from the stool collected from children at approximately the time when they were diagnosed as autoimmune. A new approach to the analysis of metagenomic DNA is described herein which allows the statistical quantitative analysis of the functional differences between cases and controls.

The analysis methodology described herein revealed striking functional differences between cases and controls. These were seen at the levels of community processes, whole pathways as well as for individual genes. At the community level, the microbiome of the healthy children are far more functionally diverse than are the autoimmune microbiomes. For example, for nearly every major function category, as defined by the SEEDS subsystems, the relative abundance of reads was statistically higher in controls rather than cases. These major categories include amino acid metabolism, carbohydrate metabolism, RNA metabolism, DNA metabolism, cell wall and capsule proteins, nucleotides and nucleosides, cofactors and vitamins, motility and chemotaxis, nitrogen metabolism, membrane transport, phosphorous metabolism, virulence, and respiration.

The lower functional diversity in cases suggests that the case microbiomes possess more bacteria that are fastidious, requiring more nutrients in the external environment for survival and growth. If the gut epithelial layer in autoimmune children is leaky as suggested by Vaarala et al. [3], the host may be leaking more substrates into the gut than is typically seen in healthy large intestines. In contrast, the abundance of reads that map to ORFs of unknown function is statistically higher in cases than controls. So although, the vast majority of bacteria found in both control and case samples can be identified to the genus level, the case genomes are much less well characterized at the functional level. One reason for this is that there appears to be a much higher abundance of anaerobes in cases than controls. One indicator of higher anaerobicity in cases is the higher number of reads mapping to sulfur metabolism in cases. Control communities have far more genes involved in aerobic respiration while cases have far more anaerobic respiratory reductases. As anaerobic bacteria are more difficult to characterize genetically than are aerobic bacteria, it is not surprising that less is known about their biochemistry than the aerobes.

**Figure 5:**
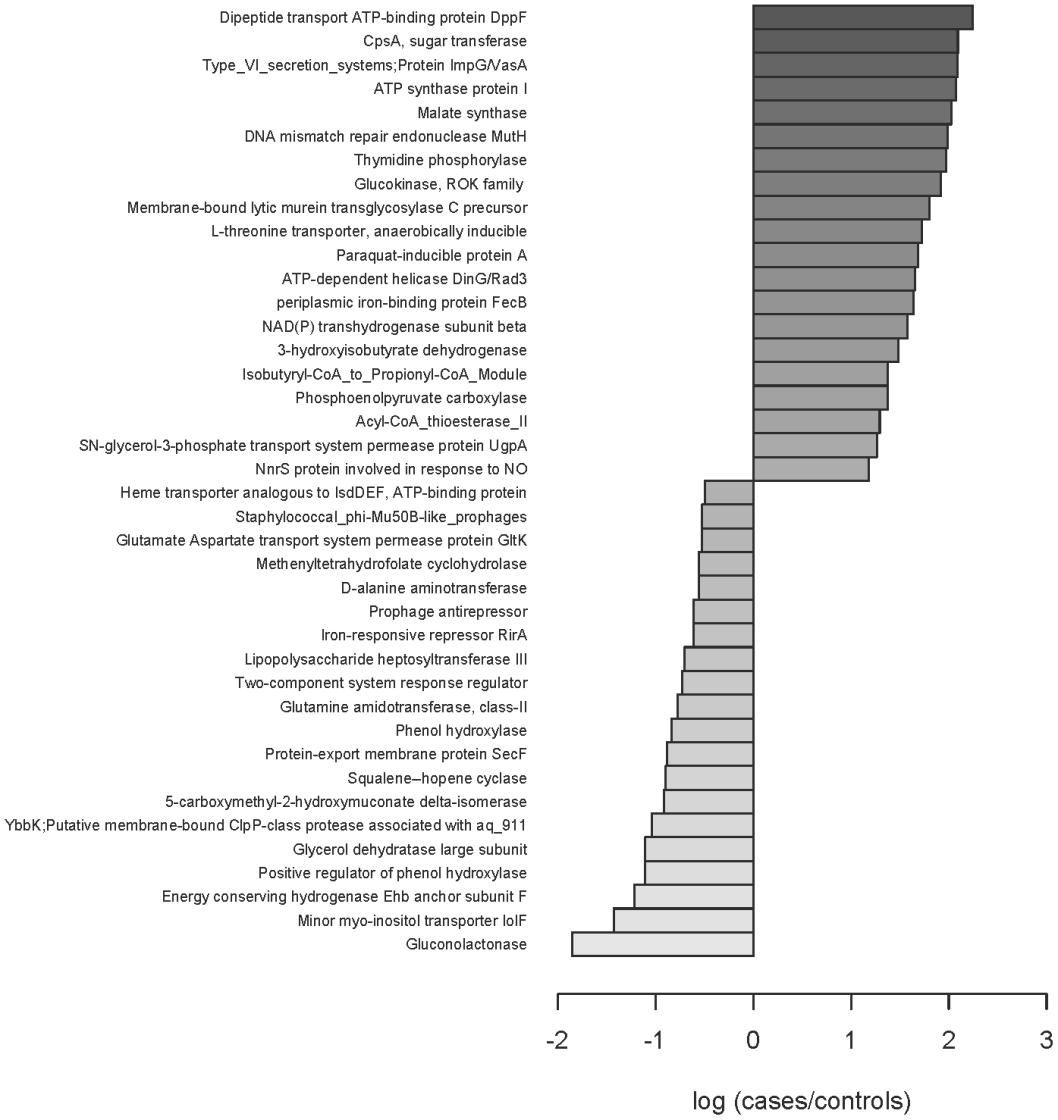
Forty known functions that differ significantly between cases and controls (p value≤0.01) as determined by the log of the ration between cases and controls. Twenty of these functions are the highest in cases relative to controls while the other twenty are the highest in controls relative to cases.

Although more reads controls are found that match known virulence determinants than in cases, there are significantly more reads in cases for some very specific virulence factors such as adhesions, *Staphylococcus* pathogenicity island genes, and antibiotic resistances. The forty most abundant differences between cases and controls show that stress responses, virulence factors, phages, prophages, quorum sensing, and motility genes are much more abundant in terms of the % of total reads in cases than controls (Fig. 5). In contrast, controls are more abundant in functions related to central metabolism such as DNA, RNA, and protein metabolism and respiration.

One of the intriguing findings with these data relates to a possible role of butyrate production in the maintenance of gut health. Butyrate is known as an anti-inflammatory short chain fatty acid that contributes to colon health [31]–[33]. The 16S rRNA mining of these data shows that many of the bacterial genera significantly more abundant in controls compared to cases are butyrate producers. In addition, butyrate induces mucin synthesis [34], [35], decreases bacterial transport across metabolically stressed epithelia [36], and improves the intestinal barrier by increasing tight junction assembly [37], [38]. Mucin is a glycoprotein made by the host that is believed to maintain the integrity of the gut epithelium. Perspective signatures of increased mucin synthesis in the gut may be the presence of *Prevotella* and *Akkermansia* as both genera are known to degrade mucin [30], [39]. *Akkermansia* and *Prevotella* are significantly more abundant in the controls than in the cases. Thus, a working hypothesis for a role for bacteria in preventing autoimmunity is that the presence of butyrate producing bacteria in healthy individuals may be inducing mucin synthesis in the gut, which maintains gut integrity. The presence of *Akkermansia* and *Prevotella* in the gut may provide a useful, simple prediction of mucin content in the gut.

**Figure 6:**
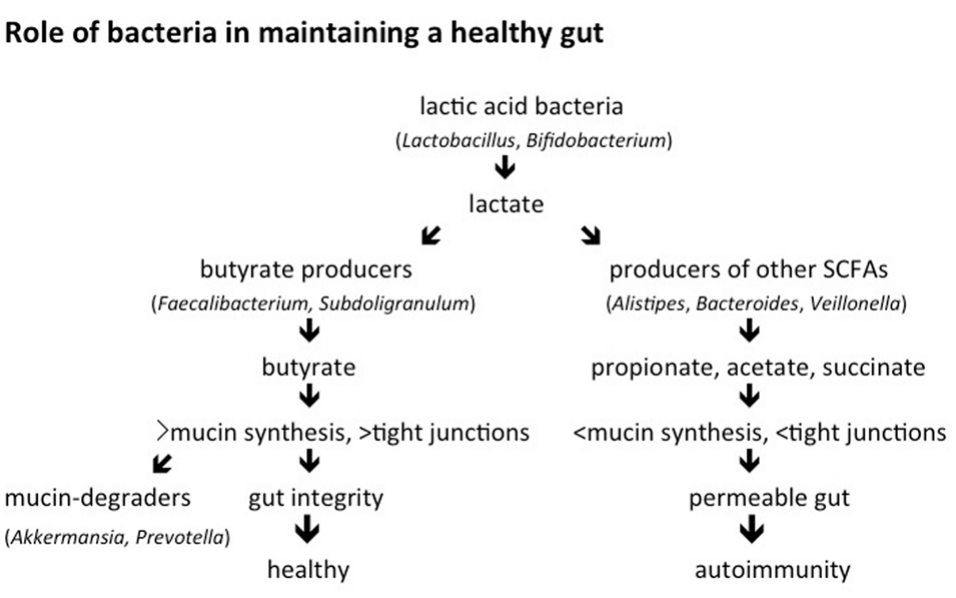
Model for a bacterial role in gut integrity leading to either a healthy state or autoimmunity for type 1 diabetes. In this model, the fate of lactate is crucial in determining gut health. Conversion to butyrate results in more mucin synthesis and tighter junctions. Conversion to other short chain fatty acids (SCHAs) reduces mucin synthesis and tight junctions. Bacterial genera listed are examples of a given phenotype. Other bacteria may also be involved in these characteristics.

All of these data, as well as work from others in the literature, suggest a model for the role of bacteria in a healthy gut (Fig. 6). The total number of lactic acid producing and butyrate producing bacteria is higher in controls than in cases [22]. Butyrate induces mucin synthesis [34], [35], [40], [41]. The higher number of butyrate producers in controls is confirmed by 16S rRNA analysis and by a higher abundance in controls of a key enzyme that catalyzes a rate-limiting step in butyrate synthesis. Mucin is well known as a glycoprotein produced by the host that contributes to gut integrity [42]. *Prevotella* and *Akkermansia* are much more abundant in controls compared to cases (Fig. 3, supporting dataset S4). These bacteria are mucin degraders [39], often found in the human gut [43], and this work suggests that they may be useful indicators of gut integrity.

In contrast to controls, the relative absence of *Prevotella* and *Akkermansia* in cases suggests a lack of mucin on the epithelial layer of intestines of cases, and may be a diagnostic of future or current gut permeability. In addition, cases have a much larger population of bacteria such as *Bacteroides*, *Veillonella* and *Alistipes* compared to controls. These bacteria ferment glucose and lactate to propionate, acetate, and succinate. Unlike butyrate, these short chain fatty acids do not induce mucin synthesis [34], [35], [40], [41]. Other factors may also be increasing mucin synthesis. Addition of a mixture of amino acids to the diets of dextran sulfate sodium-treated rats also increases mucin synthesis [44]. The control microbiomes have a significantly higher abundance of amino acid synthesis genes than do the case microbiomes.

Although lactic acid producers may be very important in the maintenance of gut health, the fate of the lactic acid produced by these bacteria may be equally important. If that fate is butyrate production, a healthy gut seems more likely. If the microbiome of the gut encourages the production of other short chain fatty acids, gut permeability may occur. Other factors also are likely to play a role in gut inflammation such as the large amount of adhesion genes found in cases. In addition, *Lactobacillus* strains can induce specific changes in the immune system of NOD mice that can increase or decrease diabetes [45]. Intestinal microbes and the innate immune system also interact to affect the development of diabetes in NOD mice [15]. Data presented here and published elsewhere suggest that microbial-induced butyrate production, and subsequent mucin synthesis, with a corresponding enhancement of tight junctions may contribute to the development of autoimmunity for type 1 diabetes in humans.

## Supporting Information

Figure S1 Average genome size in each metagenome sample as determined from the abundance of single copy, highly conserved genes in each sample. No statistical difference was observed between cases and controls.
(TIF)

Figure S2 Principle components analysis for functions shows greater similarity between controls (blue) than between cases (red).
(TIF)

Figure S3 KEGG pathways that were identified across all eight metagenomes using the SEED subsystems database.
(TIF)

Supporting Dataset S1 The percent of total reads for all statistically different functions between cases and controls are listed.
(XLSX)

Supporting Dataset S2 The percent of total reads for all functions identified in cases and controls.
(XLSX)

Supporting Dataset S3 The results of 16S rRNA mining from the metagenomic data. All six levels of taxonomic discrimination are listed with the level of statistical difference between cases and controls for each taxon. Data are listed as a % of all 16S rRNA reads mined from the metagenomic data.
(XLSX)

Supporting Dataset S4 The genera that differ statistically between cases and controls at a p-values of 0.01 or less. Data are listed as a % of all 16S rRNA reads mined from the metagenomic data.
(XLSX)

## References

[1] Karvonen M, Tuomilehto J, Libman I, Laporte R (1993). A review of the recent epidemiologic data on the worldwide incidence of type-1 (insulin-dependent) diabetes-mellitus.. Diabetologia.

[2] Patterson CC, Dahlquist G, Soltesz G, Green A, Grp EAS (2001). Is childhood-onset Type I diabetes a wealth-related disease? An ecological analysis of European incidence rates.. Diabetologia.

[3] Vaarala O, Atkinson MA, Neu J (2008). The “perfect storm” for type 1 diabetes - the complex interplay between intestinal microbiota, gut permeability, and mucosal immunity.. Diabetes.

[4] Brugman S, Klatter FA, Visser JTJ, Wildeboer-Veloo ACM, Harmsen HJM (2006). Antibiotic treatment partially protects against type 1 diabetes in the Bio-Breeding diabetes-prone rat. Is the gut flora involved in the development of type 1 diabetes?. Diabetologia.

[5] Schwartz RF, Neu J, Schatz D, Atkinson MA, Wasserfall C (2007). Comment on: Brugman S et al. (2006) Antibiotic treatment partially protects against type 1 diabetes in the Bio-Breeding diabetes-prone rat. Is the gut flora involved in the development of type 1 diabetes? Diabetologia 49 : 2105–2108.. Diabetologia.

[6] Bach JF (2002). Mechanisms of disease: The effect of infections on susceptibility to autoimmune and allergic diseases.. New England Journal of Medicine.

[7] King C, Sarvetnick N (2011). The Incidence of Type-1 Diabetes in NOD Mice Is Modulated by Restricted Flora Not Germ-Free Conditions.. Plos One.

[8] Alyanakian MA, Grela F, Aumeunier A, Chiavaroli C, Gouarin C (2006). Transforming growth factor-beta and natural killer T-cells are involved in the protective effect of a bacterial extract on type 1 diabetes.. Diabetes.

[9] Calcinaro F, Dionisi S, Marinaro M, Candeloro P, Bonato V (2005). Oral probiotic administration induces interleukin-10 production and prevents spontaneous autoimmune diabetes in the non-obese diabetic mouse.. Diabetologia.

[10] Matsuzaki T, Nagata Y, Kado S, Uchida K, Kato I (1997). Prevention of onset in an insulin-dependent diabetes mellitus model, NOD mice, by oral feeding of Lactobacillus casei.. Apmis.

[11] McInerney MF, Pek SB, Thomas DW (1991). Prevention of insulitis and diabetes onset by treatment with complete Freund adjuvant in NOD mice.. Diabetes.

[12] Sadelain MWJ, Qin HY, Lauzon J, Singh B (1990). Prevention of type-1 diabetes in NOD mice by adjuvant immunotherapy.. Diabetes.

[13] Sadelain MWJ, Qin HY, Sumoski W, Parfrey N, Singh B (1990). Prevention of diabetes in the BB rat by early immunotherapy using Freund adjuvant.. Journal of Autoimmunity.

[14] Valladares R, Sankar D, Li N, Williams E, Lai KK (2010). Lactobacillus johnsonii N6.2 Mitigates the Development of Type 1 Diabetes in BB-DP Rats.. Plos One.

[15] Wen L, Ley RE, Volchkov PY, Stranges PB, Avanesyan L (2008). Innate immunity and intestinal microbiota in the development of Type 1 diabetes.. Nature.

[16] Yadav H, Jain S, Sinha PR (2007). Antidiabetic effect of probiotic dahi containing Lactobacillus acidophilus and Lactobacillus casei in high fructose fed rats.. Nutrition.

[17] Roesch LFW, Lorca GL, Casella G, Giongo A, Naranjo A (2009). Culture-independent identification of gut bacteria correlated with the onset of diabetes in a rat model.. Isme Journal.

[18] Frank DN, Amand ALS, Feldman RA, Boedeker EC, Harpaz N (2007). Molecular-phylogenetic characterization of microbial community imbalances in human inflammatory bowel diseases.. Proceedings of the National Academy of Sciences of the United States of America.

[19] Dicksved J, Halfvarson J, Rosenquist M, Jarnerot G, Tysk C (2008). Molecular analysis of the gut microbiota of identical twins with Crohn's disease.. Isme Journal.

[20] Singh B, Read S, Asseman C, Malmstrom V, Mottet C (2001). Control of intestinal inflammation by regulatory T cells.. Immunological Reviews.

[21] Chow J, Mazmanian SK (2009). Getting the Bugs out of the Immune System: Do Bacterial Microbiota “Fix” Intestinal T Cell Responses?. Cell Host & Microbe.

[22] Giongo A, Gano KA, Crabb DB, Mukherjee N, Novelo LL (2011). Toward defining the autoimmune microbiome for type 1 diabetes.. Isme Journal.

[23] Roesch L, Casella G, Simell O, Krischer J, Wasserfall CH (2009). Influence of sample storage on bacterial community diversity in fecal samples.. The Open Microbiology Journal.

[24] Frank JA, Sorensen SJ (2011). Quantitative Metagenomic Analyses Based on Average Genome Size Normalization.. Applied and Environmental Microbiology.

[25] Giongo A, Davis-Richardson AG, Crabb DB, Triplett EW (2010). TaxCollector: Modifying Current 16S rRNA Databases for the Rapid Classification at Six Taxonomic Levels.. Diversity.

[26] Agresti A, Booth JG, Hobert JP, Caffo B (2000). Random-effects modeling of categorical response data.. Sociological Methodology 2000, Vol 30.

[27] McCullagh P, Nelder JA (1989). Generalized Linear Models.

[28] McCulloch CE, Searle SR (2001). Generalized, Linear, and Mixed Models.

[29] Fischer CR, Tseng HC, Tai M, Prather KLJ, Stephanopoulos G (2010). Assessment of heterologous butyrate and butanol pathway activity by measurement of intracellular pathway intermediates in recombinant Escherichia coli.. Applied Microbiology and Biotechnology.

[30] Wright DP, Knight CG, Parker SG, Christie DL, Roberton AM (2000). Cloning of a mucin-desulfating sulfatase gene from Prevotella strain RS2 and its expression using a Bacteroides recombinant system.. Journal of Bacteriology.

[31] Hamer HM, Jonkers DMAE, Venema K, Vanhoutvin SALW, Troost FJ (2008). Review article: the role of butyrate on colonic function.. Aliment Pharmacol Ther.

[32] Louis P, Flint HJ (2009). Diversity, metabolism and microbial ecology of butyrate-producing bacteria from the human large intestine.. Fems Microbiology Letters.

[33] Pryde SE, Duncan SH, Hold GL, Stewart CS, Flint HJ (2002). The microbiology of butyrate formation in the human colon.. Fems Microbiology Letters.

[34] Burger-van Paassen N, Vincent A, Puiman PJ, van der Sluis M, Bouma J (2009). The regulation of intestinal mucin MUC2 expression by short-chain fatty acids: implications for epithelial protection.. Biochemical Journal.

[35] Finnie IA, Dwarakanath AD, Taylor BA, Rhodes JM (1995). Colonic mucin synthesis is increased by sodium-butyrate.. Gut.

[36] Lewis K, Lutgendorff F, Phan V, Soderholm JD, Sherman PM (2010). Enhanced Translocation of Bacteria Across Metabolically Stressed Epithelia is Reduced by Butyrate.. Inflammatory Bowel Diseases.

[37] Peng LY, He ZJ, Chen W, Holzman IR, Lin J (2007). Effects of butyrate on intestinal barrier function in a Caco-2 cell monolayer model of intestinal barrier.. Pediatric Research.

[38] Peng LY, Li Z, Green RS, Holzman IR, Lin J (2009). Butyrate enhances the intestinal barrier by facilitating tight junction assembly via activation of AMP-activated protein kinase in Caco-2 cell monolayers.. Journal of Nutrition.

[39] Derrien M, Vaughan EE, Plugge CM, de Vos WM (2004). Akkermansia muciniphila gen. nov., sp nov., a human intestinal mucin-degrading bacterium.. International Journal of Systematic and Evolutionary Microbiology.

[40] Barcelo A, Claustre J, Moro F, Chayvialle JA, Cuber JC (2000). Mucin secretion is modulated by luminal factors in the isolated vascularly perfused rat colon.. Gut.

[41] Shimotoyodome A, Meguro S, Hase T, Tokimitsu I, Sakata T (2000). Short chain fatty acids but not lactate or succinate stimulate mucus release in the rat colon.. Comparative Biochemistry and Physiology a-Molecular and Integrative Physiology.

[42] McGuckin MA, Linden SK, Sutton P, Florin TH (2011). Mucin dynamics and enteric pathogens.. Nature Reviews Microbiology.

[43] Derrien M, Collado MC, Ben-Amor K, Salminen S, de Vos WM (2008). The mucin degrader Akkermansia muciniphila is an abundant resident of the human intestinal tract.. Applied and Environmental Microbiology.

[44] Faure M, Mettraux C, Moennoz D, Godin JP, Vuichoud J (2006). Specific amino acids increase mucin synthesis and microbiota in dextran sulfate sodium-treated rats.. Journal of Nutrition.

[45] Lau K, Benitez P, Ardissone A, Wilson TD, Collins EL (2011). Inhibition of Type 1 Diabetes Correlated to a Lactobacillus johnsonii N6.2-Mediated Th17 Bias.. Journal of Immunology.

