# Supplementary figures and images for: Gut Microbiome Metagenomics Analysis Suggests a Functional Model for the Development of Autoimmunity for Type 1 Diabetes
Source: PLoS One. 2011 Oct 17;6(10):e25792. doi: 10.1371/journal.pone.0025792 (PMC3197175; doi:10.1371/journal.pone.0025792)

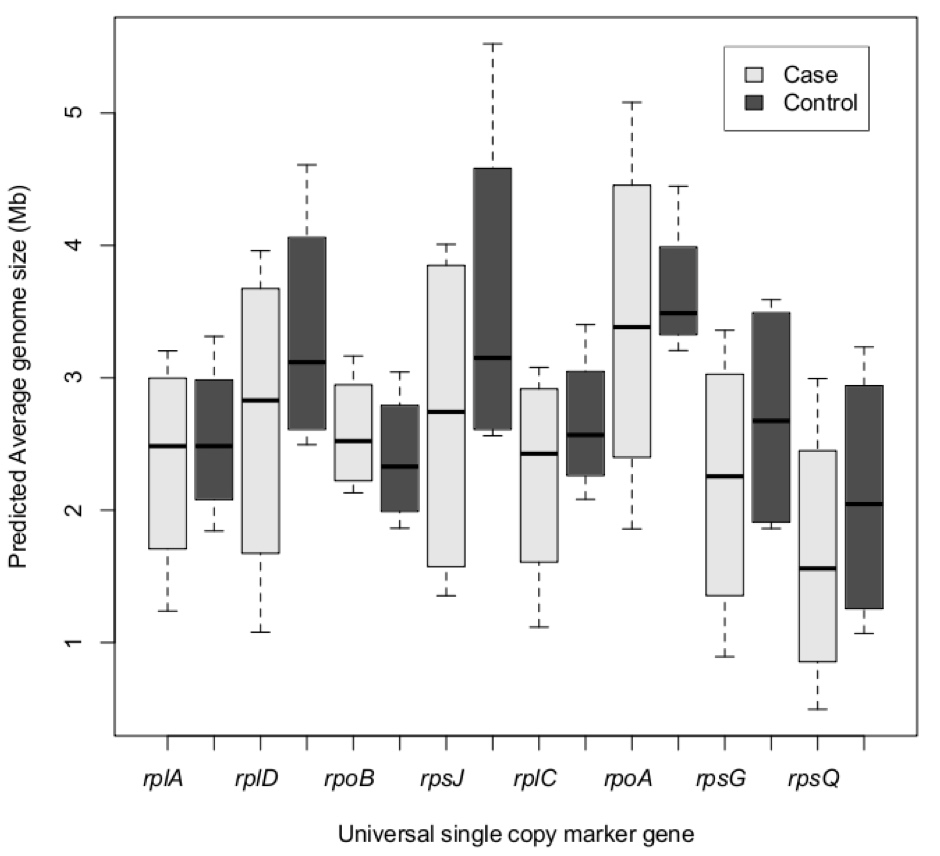

Supplement: Figure S1 — Average genome size in each metagenome sample as determined from the abundance of single copy, highly conserved genes in each sample. No statistical difference was observed between cases and controls. (TIF) [file pone.0025792.s001.tif]

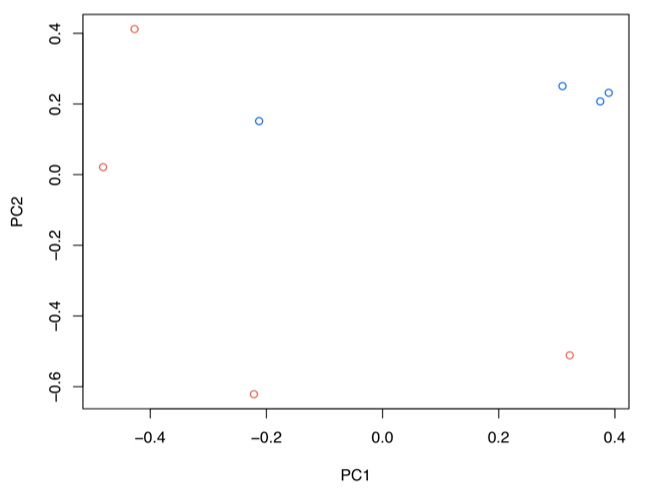

Supplement: Figure S2 — Principle components analysis for functions shows greater similarity between controls (blue) than between cases (red). (TIF) [file pone.0025792.s002.tif]

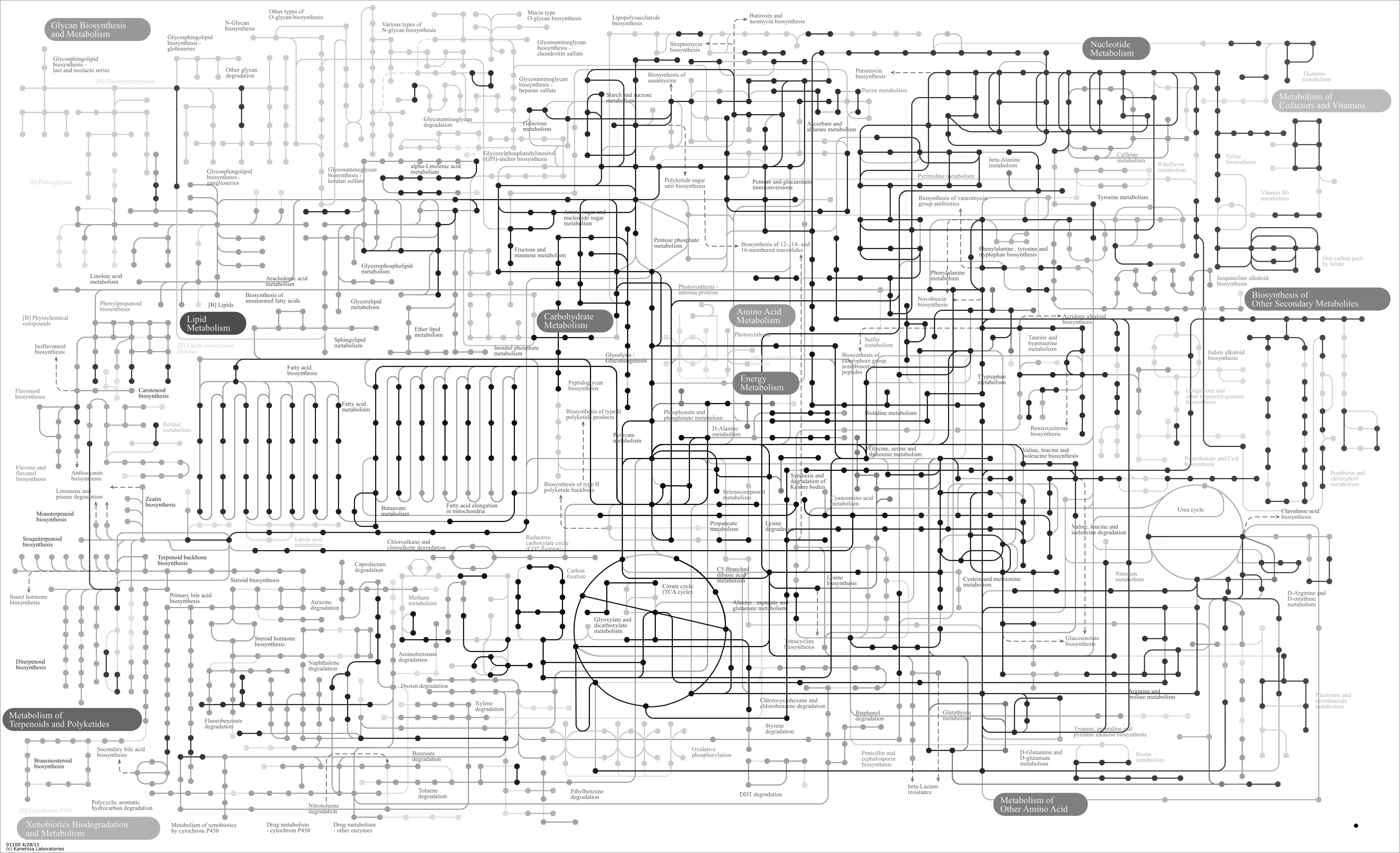

Supplement: Figure S3 — KEGG pathways that were identified across all eight metagenomes using the SEED subsystems database. (TIF) [file pone.0025792.s003.tif]
